# Reproductive Indicators, Blood Biochemistry, Steroid Hormones, and Sperm Quality of Nile Tilapia *Oreochromis niloticus* Broodstock Subjected to *Panax ginseng* Extract as Dietary Supplements During the Spawning Season

**DOI:** 10.3390/ani16213529

**Published:** 2026-11-07

**Authors:** Rasha K. Abdel-Wahed, Hamed H. E. Saleh, Gad H. G. Hussein, Abdelwahab A. Abdelwarith, Elsayed M. Younis, Simon J. Davies, Mohamed F. Abdel-Aziz

**Affiliations:** 1 Department of Animal Production, Faculty of Agriculture, Cairo University, Giza 12613, Egypt; rashakhaled123@agr.cu.edu.eg (R.K.A.-W.); gadseafox@agr.cu.edu.eg (G.H.G.H.); 2 Aquaculture Division, National Institute of Oceanography and Fisheries (NIOF), Cairo 11516, Egypt; 3 Department of Zoology, College of Science, King Saud University, P.O. Box 2455, Riyadh 11451, Saudi Arabia; awarith@ksu.edu.sa (A.A.A.); emyounis@ksu.edu.sa (E.M.Y.); 4 Aquaculture Nutrition Research Unit ANRU, Carna Research Station, Ryan Institute, College of Science and Engineering, University of Galway, H91V8Y1 Galway, Ireland; sjdplymouth@live.co.uk; 5 Department of Aquaculture and Biotechnology, Faculty of Aquaculture and Marine Fisheries, Arish University, Arish 45511, Egypt

**Keywords:** *Panax ginseng* extract, fry production, sexual hormones, semen quality, Nile tilapia Broodstock

## Abstract

To achieve sustainable aquaculture and enhance the reproductive efficiency of tilapia fish safely, our work studied the beneficial impact of a dietary extract of *Panax ginseng* (PGE) on the reproductive efficiency of Nile tilapia broodstock and the performance of their fry. The broodstock were conditioned for 21 days by separately rearing the females and males into four groups of each and fed with four levels of dietary PGE (0, 0.5, 1, and 1.5 g/kg). Following this, a 100-day spawning experiment was conducted in 12 hapas (28 m^3^) with a sex ratio of 3:1 [90 females: 30 males/hapa]. A hapa was considered an experimental unit with 3 hapas per treatment. Males and females that received the same diets in the conditioning stage were mated and continued to be fed the same feed during this stage. Hapas were checked every 10 days to collect yolk sac larvae or eggs, which were transferred to incubators. A sample of 10 males and 30 females was taken after 30 days from each group to evaluate semen characters, blood indices, the gonadosomatic index GSI, and egg diameter. At the end, a blood sample was collected from the broodstocks of each group to evaluate their biochemicals and sexual hormones. Another trial was performed for 28 days to compare the performance of the offspring from each treatment at a density rate of 20,000 larvae/hapa (12 m^3^) with a basal diet containing 40% CP. Results showed a significant increase in semen quality, sexual hormones, the GSI, hatching rate, and fry production in the broodstock fed the dietary PGE compared to the control group. Specifically, the group that received 1.5 g/kg showed the best results and the highest values in all parameters as sperm motility (92.50%), egg diameter (1.66 mm), and fry number per hapa per collection (16,515 fry) followed by those that received 1.0 and 0.5 g/kg PGE, respectively.

## Simple Summary

To achieve sustainable aquaculture and enhance the reproductive efficiency of tilapia fish, safety must rely on using natural products as feed or water additives and researching their application based on their bioactive compounds. *Panax ginseng* extract affects sexual performance and increases fertility by stimulating sex hormones, thereby enhancing fish reproductive efficiency. The present work showed that using *Panax ginseng* extract as a supplemented feed at a dose of 1.5 g/kg diet during the spawning season of Nile tilapia boosts reproductive efficiency, new hatchlings’ performance, and supports sustainability.

## 1. Introduction

Fish reproduction and sustainability are closely linked and essential to preserving aquatic ecosystems and ensuring the long-term survival of fish populations. Genetics, diet, and contemporary farming methods are some of the variables that affect the quality of fish seeds [1]. The need for premium fish seeds has compelled research into a number of methods to boost fertility in order to satisfy growing demand [2]. Functional feeds are the best, as they boost the fish’s physiological and natural health status [3]. Also, nutrition is the primary factor in maintaining the productive and reproductive performance of fish [4,5]. The use of natural phytochemicals in aquaculture has drawn more interest because of its potential to enhance fish health, development, and reproductive function [6,7,8]. Numerous plant species have been demonstrated to enhance sexual performance and encourage sexual arousal, as in the case of the female African catfish broodstock, where nutritional treatment with Garcinia kola seed extract increased fertility and gonadal weight [9]. Work has also been done on *Vitex agnus-castus*’s impact on reproduction in *Xiphophorus helleri*, feed supplementation at 1250 mg/kg dramatically increased fertility, gonadosomatic index (GSI), and larval output, according to [10]. Additionally, at a 15 g/kg inclusion level, feminization effects were observed in zebrafish larvae [11].

Previous research has demonstrated that antioxidant substances may act directly or indirectly to increase fertility, and the majority of antioxidant-rich plants may improve sperm motility and count as well as increase the formation of testosterone and estrogen [12,13,14,15]. Also, the water-based extract of *Piper guineense*, have been shown to promote fertility in both humans and animals by boosting serum and testicular testosterone levels as well as testicular cholesterol [16]. In the same text, ginsenosides, nitrogenous compounds, carbohydrates, phytosterol, organic acids, essential oils, amino acids, and peptidoglycans are among the many phytochemicals found in *P. ginseng* [17]. *Panax ginseng* is the most commercially available species; it has been used as a growth promoter, immune stimulator, anti-inflammatory, antidiabetic, antitumor, anti-obesity, cardioprotective, antimicrobial, neuroprotective, aphrodisiac, and antioxidant [18]. Numerous useful bioactive substances, including alkaloids, phenolics, vitamins, amino acids, proteins, ginsenosides, and polysaccharides, are mostly found in *Panax ginseng* [19]. Ginsenosides have potent anti-inflammatory, anti-allergic, antidiabetic, and antioxidant properties [20]. Accordingly, their antioxidant properties increase fertility by protecting reproductive organs from inflammation, supporting reproductive health [21].

In this regard, ginseng’s application in aquaculture seems promising. For example, dietary *Panax ginseng* polysaccharides have been shown to boost and enhance African catfish reproductive efficiency [22]. Furthermore, *Panax ginseng* affects sexual effectiveness and increases fertility by influencing sex hormones and their receptors [23,24]. Despite the positive results associated with the ginseng diets mentioned earlier, limited information is available regarding the effects of dietary *Panax ginseng* extract (PGE) on Nile tilapia (*Oreochromis niloticus*) broodstock reproductive performance. Several factors affect its effectiveness in enhancing fish reproductive efficiency, including dosage, rearing conditions, and the duration of feeding. It is believed that providing ginseng during the stock preparation period and continuing its use during spawning can have beneficial effects on reproductive efficiency and improve fry performance after hatching. This approach presents an environmentally friendly method to promote sustainable food production. Therefore, the present study aimed to use *Panax ginseng* extract (PGE) as a feed additive in *O. niloticus* broodstock and evaluate its role on reproduction indices and the performance of their fry.

## 2. Materials and Methods

### 2.1. Workplace and the Ethics Declaration

This work took place on a commercial farm in Fayoum Governorate, Egypt, to evaluate four dosages of *Panax ginseng* extract (PGE) as supplements in broodstock diets, emphasizing their impact on reproductive efficiency and the growth performance of newly hatched offspring over a period of 28 days. All procedures used in this study, encompassing rearing, handling, sampling, and euthanasia, adhered to the guidelines established by the Committee for Ethical Care and Use of Animals/Aquatic Animals of the National Institute of Oceanography and Fisheries (NIOF, Egypt), which accepted this study within the code (NIOF-IACUC, Code: NIOF-AQ1-F-25-R-029). Additionally, experimental techniques followed the pertinent standards and regulations in compliance with the ARRIVE criteria.

### 2.2. Getting the Bloodstock Flock Ready for the Spawning Season

A 21-day preparation period preceded the spawning, during which broodstock were kept separately in eight hapas (four for males and four for females) measuring 7 m × 4 m × 1.5 m (L × W × D), placed in an earthen pond, and equipped with a 2 HP blower. There were 270 females per hapa and 90 males per hapa. Hapas for both males and females were numbered from one to four and each hapa was given a diet (30%) that included varying amounts of *P. ginseng* extract PGE (0, 0.5, 1, and 1.5 g per kilogram for hapas 1, 2, 3, and 4, respectively). The broodstock during this period were fed at a rate of 4% of biomass. Following this period, the spawning experiment was conducted in which spawning occurred in a pre-made earthen pond (0.5 acres with a 2 HP blower), where 12 spawning hapas were set up with a water depth of 0.9 m.

### 2.3. Spawning Experiment

**Figure 1:**
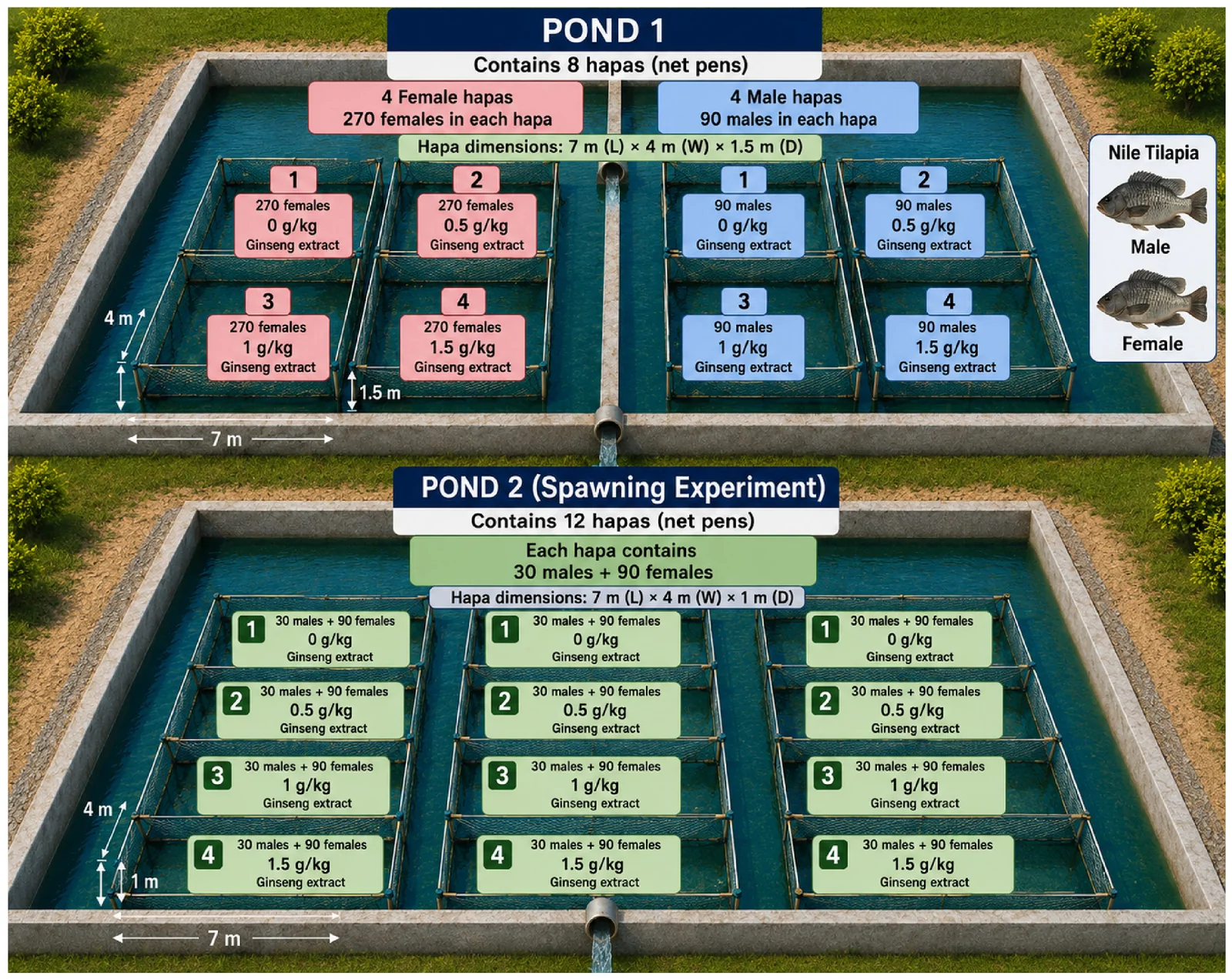
Experimental design and fish distribution in both stages: pre-spawning (preparation) and spawning.

The 12 hapas measured 7 m × 4 m × 1 m (L × W × D) and were randomly stocked with females and males that were fed with the same diet during the preparation period at a rate of 120 broodstock per hapa (90 females: 30 males; sex ratio 3:1). Broodstock that were healthy, ripe, and of similar size were selected to prevent male violence during spawning. Male and female average weights ranged from 250 to 270 g and 200 to 225 g, respectively, at one year of age. Four experimental doses of *P ginseng* extract per kilogram of basal diet were included in the experimental design during the spawning stage, which was a continuation of the same dietary treatments throughout the conditioning period (PGE0: control group; PGE0.5, PGE1, and PGE1.5, respectively). The trial lasted 100 days, and each treatment was replicated (three hapas per treatment group Figure 1).

### 2.4. Feed and Feeding Managements

As shown inTable 1, a basal feed with 30% crude protein was formulated using fish feed, gluten corn, yellow corn, wheat bran, and soybean meal. The feed ingredients were thoroughly ground and mixed, after which the experimentally determined doses of PGE, loaded onto carboxymethyl cellulose (a primary component of the basal diet) to ensure thorough mixing and increased homogeneity, were added. Each experimental feed mixture was then moistened with a small amount of water to facilitate pelleting, which was conducted using a meat grinder fitted with a 4 mm die. The pellets were sun-dried and stored at 4 °C. Broodstock were manually fed twice a day, six days a week, at 9 a.m. and 4 p.m., at a level of 1% body weight according to [25]. Additionally, broodstock were starved on the days on larva collection days. The water exchange was 30% of the total volume every 3 days. The PGE used was a commercial product, red *Panax ginseng* extract VEG capsules (KOREAN PANAX GINSENG, BLLIS OF EARTH) in a bottle of 60 capsules; each capsule contained 500 mg of extract, manufactured by Harmony Lifesciences.

*Panax ginseng* extract is characterized by a complex chemical composition; major ginsenosides reported in red ginseng extracts include protopanaxtriols, with an approximate HPLC analysis of [Rg1(0.30–2.33 mg/g); Re 0.90–1.80 mg/kg); Rf (0.83–1.1 mg/g)] and protopanaxdiols [Rb1 (5–7.80 mg/g), Rb2 (1.80–2.94 mg/g), Rc (1.95–3.15 mg/g), Rd (0.60–1.28 mg/g)] as well as non-saponin constituents including polysaccharides, amino acids, peptides, and phenolic compounds [26,27].

**Table 1:** Formulation of the basal diet and the approximate analysis of chemical composition.

Ingredients (%)	Basal Diet
Fish meal	12
Corn gluten	6
Meal of soybean	32
Corn (yellow)	28
Bran of wheat	15
Soy oil	5
^1^ Vit. & Min.	1
^2^ CMC	1
^3^ Chemical compositions (%)	
Dry matter	90.35
Protein	29.86
Ether extract	8.78
Fiber	5.35
Ash	6.25
^4^ NFE	40.11
^5^ Gross energy, MJ/kg	18.32

^1^ Each vitamin mix (3 Kg) contains: 1200 000 IU, 300 000 IU, 700 mg, 500 mg, 500 mg, 200 mg, 600 mg, 3 mg, 450 mg, 3000 mg, 3000 mg, 10 000 mg, 300 mg, 6 mg, and 670 mg, forvitamins of A, D3, E, K3, B1, B2, B6, B12, C, Niacin, Methionine, Cholin chloride, Folic acid, Biotin, and Panthonic acid respectively. Each mineral mix (1 Kg) contains: 1472 mg, 1030 mg, 2359 mg, 747 mg, 5 mg, 33 mg, 1.28 mg, 4300 mg 32.37%, 4000 mg, 52% for Manganese Sulphur, Zinc Sulphur, Iron Sulphur, Copper Sulphur, Cobalt Sulphur, Potassium iodide, Sodium selenite, Sodium Sulphur, and Potassium chloride respectively. ^2^ CMC: Carboxy methyl cellulose. ^3^ Diets were analyzed using the methods described by [28]. ^4^ Calculated by difference: 100 − (lipid+ protein+ fiber+ ash). ^5^ Calculated using gross calorific values of 23.63, 39.52 and 17.15 kJ/g for protein, fat and carbohydrate, respectively, according to [29].

### 2.5. Eggs Hatching and Fry Harvest

Hapas were checked every 10 days to collect the mouthbrooded eggs or newly hatched. Fertilized eggs were taken to a hatching laboratory equipped with eight incubator jars (15 L), two of which were assigned to each treatment to make it easier to calculate the hatching rate of each treatment. Meanwhile yolk sac larvae were collected using a fine scoop net, transferred to nursery hapas, and fed a diet containing 17-alpha methyltestosterone (80 mg/kg fine diet—40% CP) for 28 days.

### 2.6. Criteria of Water Quality

Two locations at equal distances were identified within each hapa to collect water samples. A multi-parameter water quality meter, model BEP-M903, was used daily to measure water temperature, pH, and soluble oxygen (DO). Alkalinity, total ammonia, NH_4_-NH_3_, NO_2_, and NO_3_ were determined weekly [30]. These parameters ranged from 26–32 °C, 8.15–8.25, 6–7 mg/L, 260–380 mg/L, 0.28–0.45 mg/L, 0.42–0.62 mg/L, and 4.28–6.18 mg/L, respectively. Generally, all water parameters fell within acceptable limits for tilapia growth and health [31].

### 2.7. Calculations

#### 2.7.1. Examinations of Reproductive Efficiency

The diameter of the eggs was measured from a 1 g sample obtained from the ovaries of a female, preserved in 5% formalin, and subsequently placed on a slide for examination using an eyepiece micrometer under 10× magnification.

Gonadosomatic index (GSI, %) = [gonads weight/broodstock weight] × 100.

Hatching rate (%) = [No. hatched eggs/total collected eggs] × 100.

Survival rate (%) of Broodstock = [No. of Broodstock at the end/No. of Broodstock at the start] × 100.

NO. of spawned females/hapa/period = Number of spawned females number per hapa × number of collection.

Number of spawned females (%/collection) = (NO. of spawned females/hapa)/(number of females per hapa) × 100.

Number of spawning/female/period = (NO. of spawned females/hapa per period)/(number of females per hapa).

Spawning intervals = period of experiment/number of spawning per female per period.

#### 2.7.2. Examinations of Fry Production

Number of Fry per female/collection = (Number of Fry per hapa per collection)/(Number of spawned females per hapa per collection).

Number of Fry/female/period = (Total fry production/hapa/period)/(number of females per hapa).

Feed required to produce 1000 fry = (Feed offered to broodstock per period)/(Total fry production per hapa per period) × 1000.

#### 2.7.3. Fry Performance and Survival Rate

Weight gain, mg = (final weight, g) − (initial weight, g).

Specific growth Rate (SGR), %/day = [(ln final weight-ln initial weight)/days] × 100.

Rate of feed conversion (FCR) = offered feed (W)/weight gain (W).

Survival rate, % = [No. of fry at the end/No. of fry at the start] × 100.

### 2.8. Sampling

Thirty days post-spawning, following a 24 h fasting interval, a random sample of broodstock (9 males and 27 females/treatment or 3 males and 9 females/hapa) was collected for the analysis of blood biochemistry, blood sex hormones, semen quality, the gonadosomatic index, and egg diameter. Before blood collection, the fish were anesthetized in a water bath using tricaine methane sulfonate (MS-222) at a dose of 100 mg/L [32]. After blood sampling, the anesthetized fish were humanely euthanized through decapitation. Then, the gonads, which include the ovaries and testes, were collected for analysis, and measurements of reproductive efficiency were taken.

This procedure was repeated at the end of the experiment (after 100 days) using the same sample size (3 males and 9 females from each hapa), with the process limited to the collection of blood samples only. After blood collection, the anesthetized fish were returned to their hapas once they had recovered. Blood samples were collected from the caudal veins into syringes (3 ml) without anticoagulants. For half an hour, the samples were left to coagulate at 4 °C. The serum was then separated for biochemical analysis by centrifugation for 15 min at 4000× *g*.

#### 2.8.1. Blood Analyses

Serum levels of estrogen (Cat. No: CSB-E13017Fh), progesterone (Cat. No: 11-PROHU-E01), and testosterone (Cat. No: MBS933475) were measured using commercial ELISA kits [33]. According to [34] the cortisol was spectrophotometrically determined by ELISA method using a kit (DRG Cortisol ELISA EIA-1887, Germany). Cholesterol and triglyceride levels were measured using commercial test kits, following the methodology outlined by [35].

#### 2.8.2. Semen Criteria

The males’ testes were collected by opening their abdomens. The sperm were then removed from the testes using a surgical cutter, and the contents were emptied into Bendform tubes (2.0 mL) and carefully identified for assessing the semen. For microscopic counting, semen was diluted 1:4 with 0.9% NaCl using a hemocytometer [36,37,38].

A 2 µL aliquot was placed on a clean microscope slide and activated by adding 100 µL of distilled water, following the approach reported for Nile tilapia by [39]. Motility was examined within 20–40 s after activation at a controlled temperature of 25 ± 1 °C using a light microscope at 100× total magnification, provided by a 10× objective and a 10× eyepiece. Five non-overlapping microscopic fields were examined, and at least 200 spermatozoa were evaluated across the fields in each of three technical replicate observations. Spermatozoa were classified as rapidly progressive, slowly progressive, non-progressive, or immotile [40]. Simultaneously, the following indicators were estimated: morphology was assessed by preparing the fixed sperm suspension as a smear and staining it with Bengal rose dye. A total of 200 spermatozoa per sample were examined under a light microscope at 100× magnification. Spermatozoa exhibiting an intact head, mid-piece, and flagellum were classified as morphologically normal. Morphological abnormalities were expressed as a percentage of the total number of spermatozoa examined [39].

### 2.9. Growth and Survival Rate of New Hatchlings

To assess the long-term impacts of broodstock PGE supplementation on the performance and survival rate of yolk sac fry over a period of four weeks, newly hatched fry resulting from each treatment group were placed into three hapas (an experimental unit with dimensions: 4 m × 2 m × 1 m; length × width × depth), meaning 12 hapas for all groups, which were set up in a separate earthen pond. The fry were transferred from incubator jars to the nursery hapa 24 h after hatching, with a stocking rate of 20,000 larvae per hapa. These fry were hand-fed the following day with a basal diet consisting of 40% crude protein (CP), supplemented with 17-alpha methyltestosterone. The feeding rate gradually decreased over time, starting at 20% during the first week, then reducing to 15% in the second week, and finally reaching 10% until the end of the experiment. A regular feeding schedule was followed, with five feedings each day, at a rate of one meal every two hours between 9 a.m. and 4 p.m.

### 2.10. Data Analysis

As aforementioned, this study includes four dietary treatments; each dietary treatment consisted of three independent hapas, representing three independent experimental replicates (*n* = 3 per treatment). Before statistical analysis, the normality of the model residuals was assessed using the Shapiro–Wilk test, together with visual inspection of normal Q–Q plots. Homogeneity of variances among experimental groups was assessed using Levene’s test. A *p*-value greater than 0.05 indicated no statistically significant evidence against the corresponding assumption. The assumptions of normality and homogeneity of variance were considered acceptable; following the completion of the data’s normality test, the mean variances were compared using Tukey’s B comparison test after conducting one-way analysis of variance (ANOVA). The results are presented as averages, and *p* ≤ 0.05 was deemed significant. All statistical procedures were completed using SPSS software, version 18.0 (SPSS Inc., Chicago, IL, USA).

## 3. Results

### 3.1. Reproductive Traits of Broodstocks

**Figure 2:**
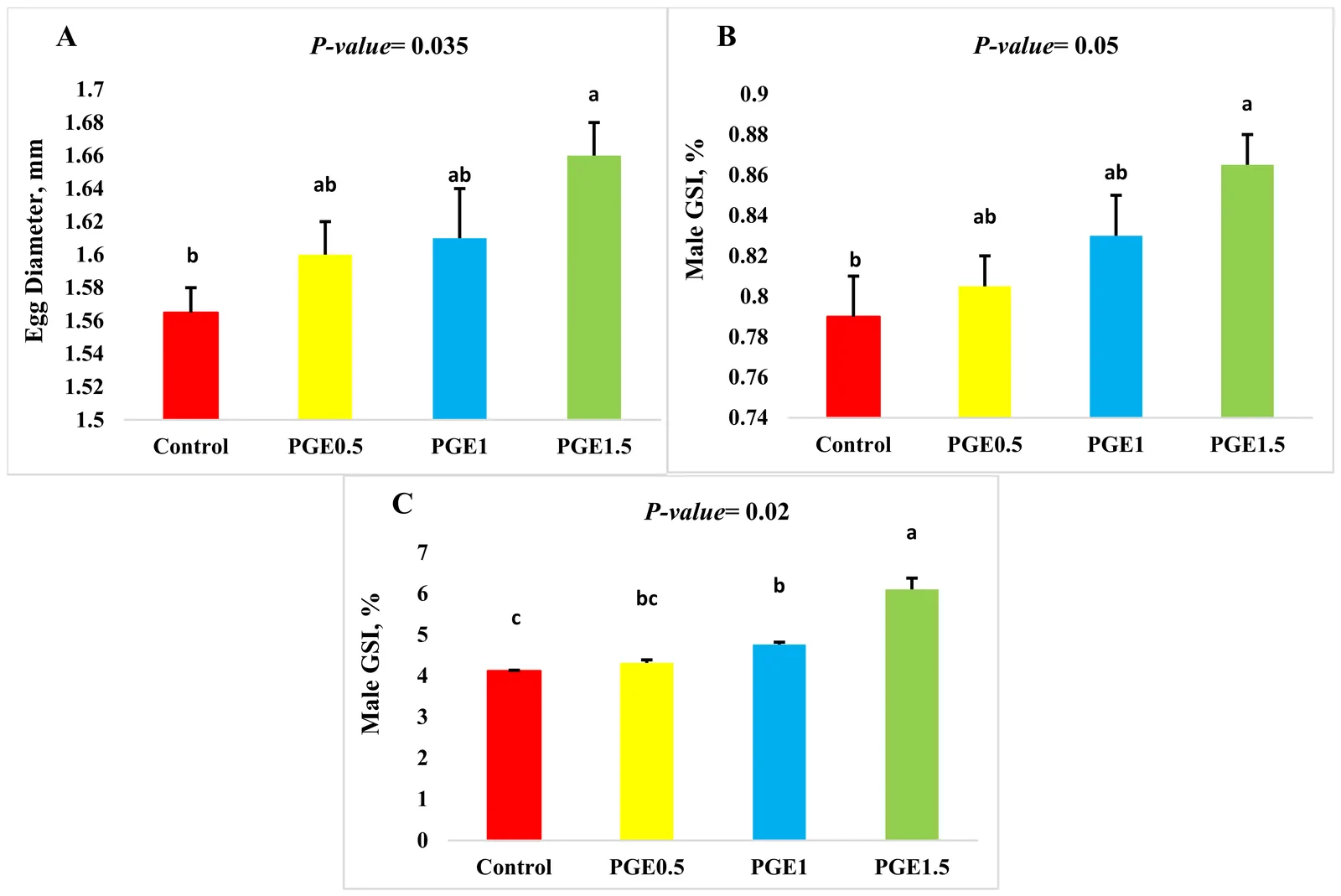
(**A**–**C**): Egg diameters and GSI of *Oreochromis niloticus* broodstock fed different doses of PGE-supplemented feed 30 days after starting the spawning experiment. Significant differences are indicated by different lowercase letters (*p* ≤ 0.05). PGE0.5, PGE1, and PGE1.5 are the tested doses of 0.5, 1, and 1.5 g of *Panax ginseng* extract per kg of diet. Error bars represent SE.

**Figure 3:**
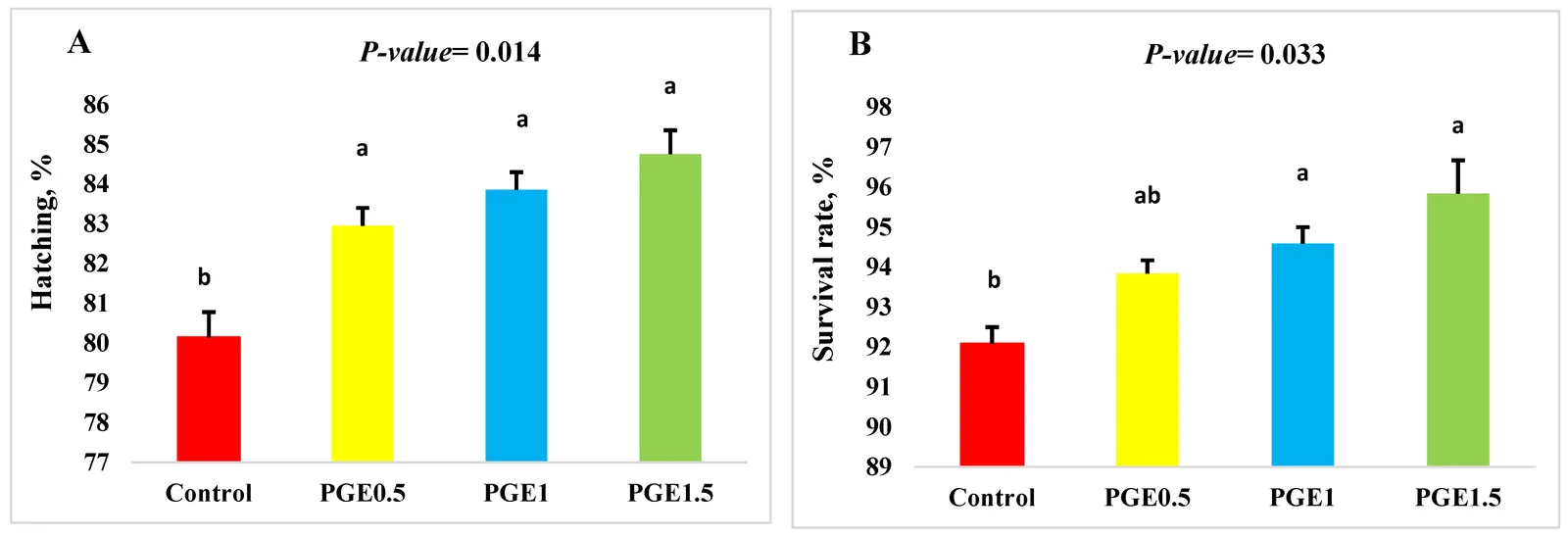
(**A**,**B**): Hatching and survival rates of *Oreochromis niloticus* broodstock fed different doses of PGE-supplemented feed for 100 days. Significant differences are detected across means with different lowercase letters (*p* ≤ 0.05). PGE0.5, PGE1, and PGE1.5 are the tested doses of 0.5, 1, and 1.5 g of *Panax ginseng* extract per kg of diet. Error bars represent SE.

The results in Figure 2A–C indicated that egg diameter and the GSI of males and females were significantly affected by dietary PGE and gradually increased with increasing PGE levels. Groups fed dietary PGE1.5 and PGE1 recorded the highest mean values for these indicators, followed by dietary PGE0.5, while the control group showed the lowest values. A similar trend was noted in the hatching rate and survival rate of broodstock, as shown in Figure 3A, B.

### 3.2. Semen Quality

Dietary PGE had a significant effect on semen quality, and there were gradual enhancements with increased doses of PGE (Table 2). Males administered dietary PGE 1.5 had the highest sperm count, motility, forward motility, and sluggish motility (%), followed by the PGE 1 and 0.5 groups, respectively. However, the control group had the highest immotile percentage.

### 3.3. Serum Biochemical Assay

The blood biochemistry of females 30 days after starting the spawning experiment and at the end is presented in Table 3. There was a significant decrease in cortisol, triglycerides, cholesterol, and estrogen levels with increasing doses of dietary PGE. The control group exhibited the highest concentrations of these parameters, while the dietary PGE1.5 group showed the lowest values. Conversely, the PGE1.5 and PGE1 groups recorded the highest progesterone concentrations, followed by the PGE0.5 group, while the control group had the lowest values.

The blood parameters of males 30 days after starting the spawning experiment and at the end (Table 4) showed an insignificant variation in cortisol; however, the control possessed the highest cortisol level, followed by the PGE0.5 and PGE1 groups, with the lowest values recorded in the PGE1.5 group. Cholesterol levels were in line with cortisol, but with significant effects among groups. Conversely, dietary PGE1.5 exhibited the highest triglyceride and testosterone concentrations, followed by the PGE1 and PGE0.5 groups, while the control recorded the lowest values.

### 3.4. Spawning Indicators

The findings in Table 5 revealed notable variations in spawned female number/hapa/period, spawned female number/hapa/collection, spawned female, %/collection, and number of spawning/female/periods. When compared to the control, the fed groups that received dietary PGE had the highest values in these parameters. Furthermore, PGE1.5 and PGE1 in the diet had the highest values, followed by PGE0.5. In contrast, the control group had the longest spawning intervals, followed by the PGE0.5, PGE1, and PGE1.5 groups, in that order.

### 3.5. Fry Production

**Figure 4:**
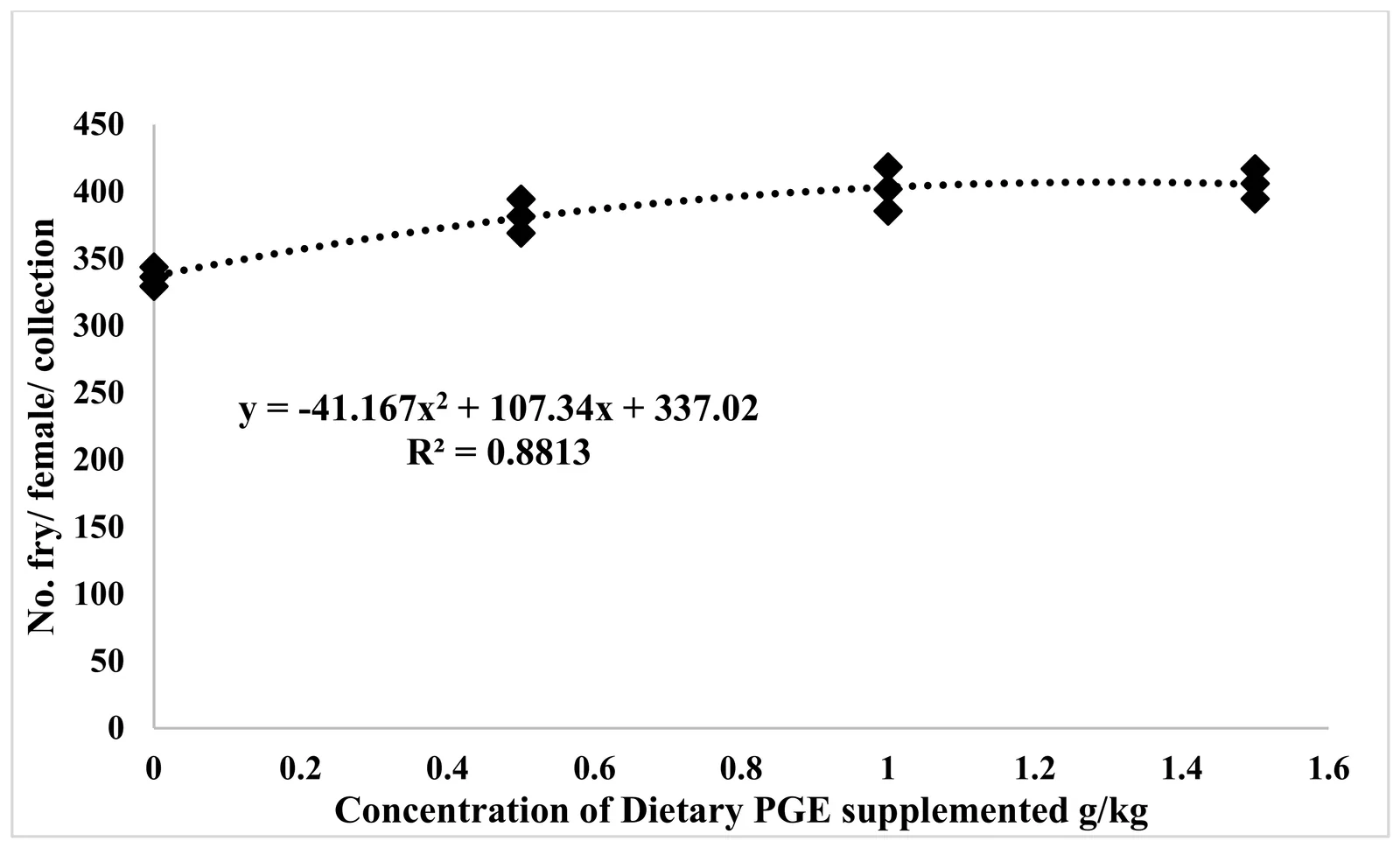
Relationship between fry No/female/collection as means of each replicate (hapa or experimental unit) and the concentrations of dietary PGE.

Results in Table 6 and Figure 4 illustrate significant improvements at *p* ≤ 0.05 in fry production indicators in treated groups fed dietary PGE compared to the control. Measurements, including fry number per hapa/collection, fry number per female/collection, fry number per female/period, total hapa production, and fry/period, were highest in the PGE1 and PGE1.5 groups, followed by the PGE0.5 group, while the control group showed the lowest values. On the other hand, the control group had the highest feed amount to produce 1000 fry.

### 3.6. Performance of New Hatchlings Larvae

Results in Table 7 present significant differences in the growth rate, feed utilization and survival rate of newly hatched larvae as a result of the effects of extended feeding of the broodstock. Larvae derived from broodstock fed dietary PGE1.5 showed the highest performance and the best FCR, followed by larvae from the PGE1 and PGE0.5 groups, while the worst performance of larvae was observed in larvae from the control broodstock.

## 4. Discussion

Medicinal herbs may stimulate fish reproduction, which would promote the sustainability of aquaculture as a result of their content of natural bioactive compounds, including sterols, flavonoids, and saponins, as well as vital minerals that contribute to their positive effects [41]. These compounds possess antioxidant properties, which directly or indirectly increase fertility by protecting reproductive organs from oxidative stress and inflammation, thereby supporting reproductive health and boosting sperm motility, morphology, and count [42,43]. Consequently, herbs can also affect testosterone production, promote spermatogenesis, and help with oocyte maturation, according to [44]. Phytochemicals, which include ginsenosides, nitrogenous substances, carbohydrates, phytosterols, organic acids, essential oils, amino acids, and peptidoglycans, are abundant in ginseng. They have notable roles as immunological modulators, growth enhancers, anti-inflammatory agents, antidiabetics, anticancer agents, anti-obesity treatments, cardioprotective substances, antibacterial agents, neuroprotective chemicals, aphrodisiacs, and antioxidants [45,46].

**Figure 5:**
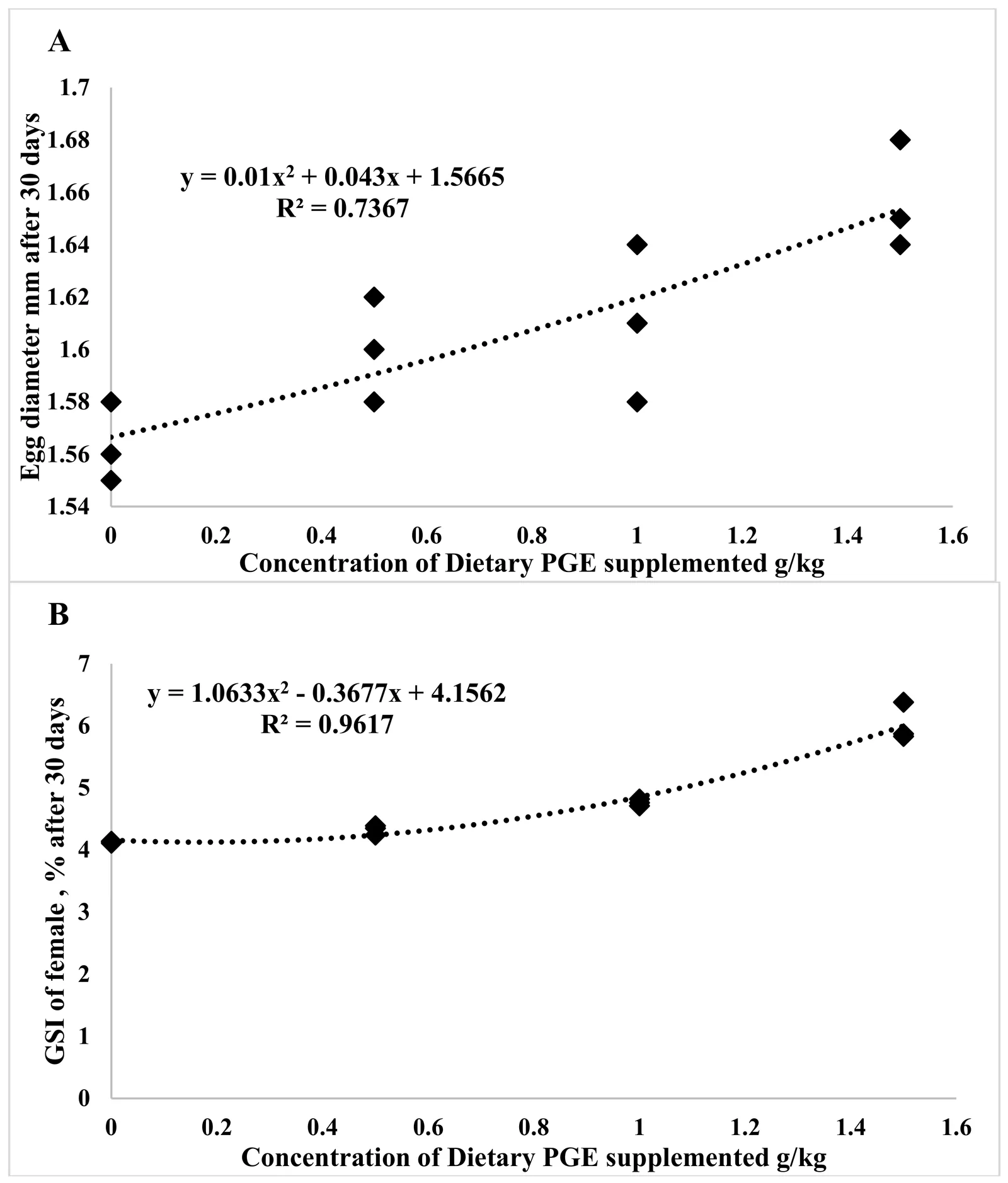
(**A**,**B**) The relationship between egg diameter and GIS of female as the mean of each replicate (hapa or experimental unit) across different dietary concentrations of PGE.

**Figure 6:**
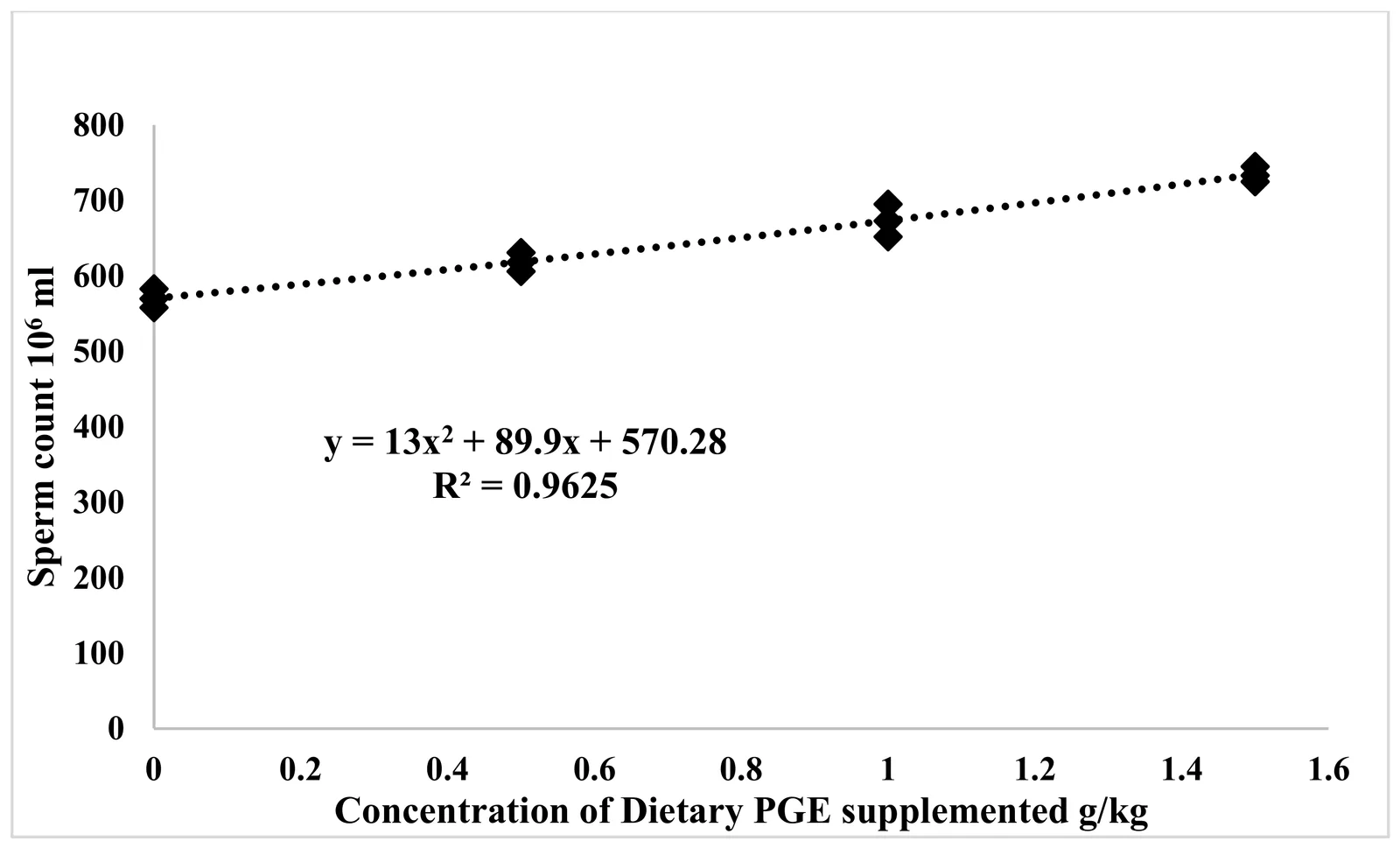
The relationship between sperm count concentrations as the mean of each replicate (hapa or experimental unit) across different dietary levels of PGE.

A previous study, Ref. [47], examined how ginseng affects rats’ and mice’s sexual behavior and performance. The authors discovered that in male rats and mice, ginseng markedly increased libido and copulatory activity. Our data affirmed that dietary PGE increased the GSI and egg size of broodstock after 30 days of starting the spawning period, and there were also improvements in semen quality. Moreover, there was a linear relationship between these parameters and the dose of dietary PGE (Figure 5A,B and Figure 6).

GSI is one of the most important biological indices for tracking changes in nutrition, energy status, and fish health. It is also a crucial metric for assessing fish fertility or spawning, because it is closely linked to the growth and quality of the gonads [48,49]. In support of these observations, a study by [22] reported that the improvement of GSI in male African catfish is the main indication that dietary ginseng enhances rates of growth and nutrient utilization. In comparison to the other treatments, they found that increasing the amount of dietary Ginseng^®^ (200 mg/kg diet) considerably enhanced the ultrastructure of spermatozoa and all the measured sperm quality indices. According to Ref. [50], adult male *Oncorhynchus mykiss* fed a diet containing 2 g of GPE per kilogram had the highest GSI value. Additionally, ginseng helps regulate body functions, specifically improving adaptability, and is believed to enhance sperm production [51]. In the same context, Ref. [52] studied the impact of ginseng on spermatozoa function in vitro. They found that ginseng at a dose of 2 mg/ml exhibited a significant protective effect by ameliorating chromatin damage. They suggested that by improving spermatozoa function, ginseng may be advantageous for reproductive function. Recent research on the impact of PGE on male reproductive function in *Oncorhynchus mykiss* showed that increasing ginseng supplementation levels improved both the semen quality and fertilization rate [50]. Furthermore, Ref. [22] suggested that ginseng provides pro-fertility qualities for African catfish, which might lead to the production of sufficient and higher-quality fish fry. Consequently, there is a high correlation between fish reproductive performance and sperm motility. According to the current study, employing PGE to boost sperm motility results in a considerably higher hatching rate and subsequently fry generation. Ginsenosides, the majority of bioactive molecules in ginseng, are responsible for the beneficial effects on reproductive performance. Ginsenosides effectively enhance the synthesis of nitric oxide (NO) ginsengting NO synthase, which in turn activates steroid receptors [53].

In relation to the effect of PGE on females, Ref. [54] showed that ginsenoside supplementation dramatically enhanced the granular cells number, a primary component of the ovarian follicle in laying hens. The granular cells in hens on the ginsenoside-supplemented diet had a more cuboidal form. This could be a main contributing factor to the increase in laying performance of the Kabir hens fed *Panax ginseng* root meal as a feed additive.

Blood biochemical indicators reflect the physiological status of fish, which can be affected by sexual maturation, age, species, diet, and the ecosystem. The physiological status of fish changes significantly during the spawning season due to the secretion of endogenous sex hormones and external stimulants [55].

**Figure 7:**
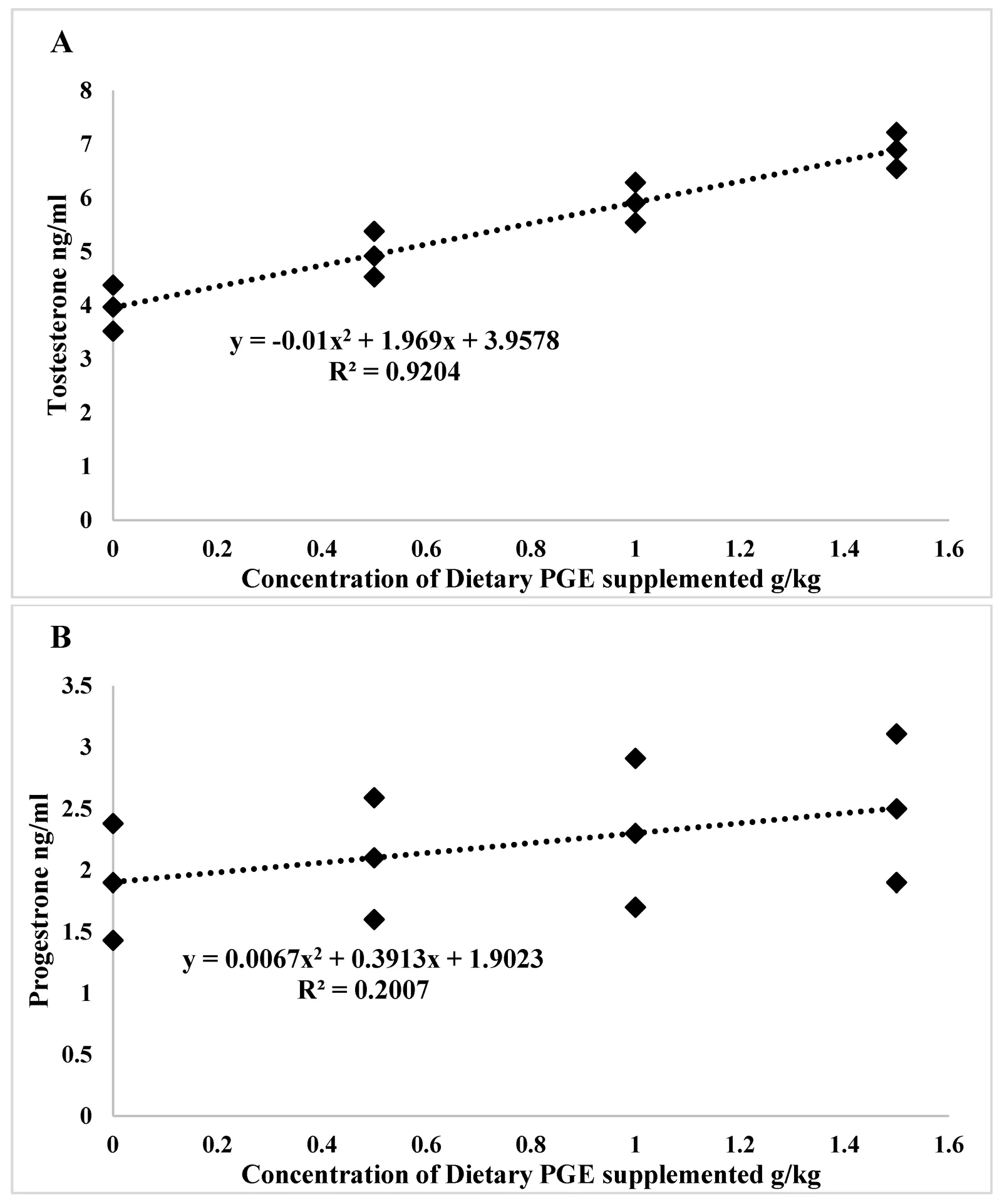
(**A**,**B**) The relationship between concentrations of testosterone and progesterone at the end of the experiment as the mean of each replicate (hapa or experimental unit) and the concentrations of dietary PGE.

Cholesterol acts as a precursor to the production of sex hormones. A variety of sex steroids are produced as a result of steroid biosynthesis, which mostly takes place in the somatic cells of the testes and ovaries [56]. By cleaving cholesterol, the process starts with the synthesis of the steroid precursor pregnenolone, which is followed by the formation of progesterone. The granulosa cells in the ovary produce thecal steroids, mostly testosterone, which are then converted to estradiol-17 h by the expression of the enzyme aromatase. Oocyte development demands the latter steroid [57]. Consequently, our data is in agreement with these findings, where dietary PGE led to decreased blood cholesterol in females and males with increased progesterone and testosterone levels. In a similar vein, zebrafish treated with 10% and 20% aqueous extracts of red ginseng extract showed a significant decrease in plasma total cholesterol levels when compared to those fed a diet high in cholesterol [24]. Accordingly, *Oncorhynchus mykiss* fed varying amounts of *P. ginseng* extract showed a non-significant drop in plasma total cholesterol when compared to the control [58]. Fish health can be accessed via the cortisol level, which is a physiological biomarker [59]. PGE dietary integration reduced cortisol, confirming its strong anti-stress effects as documented in earlier studies [60]. Ginsenosides have been shown in numerous studies to be a successful treatment for immunological and metabolic illnesses, as well as for organ damage and cell death [61]. Additionally, in vivo experiments have demonstrated that ginseng has superior anti-stress benefits when compared to the appropriate controls [62]. Regarding steroid hormones, dietary PGE enhanced testosterone in males and progesterone (Figure 7A,B) in females while also decreasing estrogen.

Generally, elevated reproductive blood hormones, such as testosterone and progesterone reflect the gonadal development and maturation of ovarian and testicular tissues in fish [63]. Because ginsenosides are triterpenoid saponins with structural similarities to steroid hormones, they may have an impact on sex hormone action and sexual function by partially agonistically binding to certain steroidal receptors [64]. Ginseng is crucial for preserving normal androgen receptor levels, where its polysaccharide content aids in promoting testosterone levels and upregulating androgen receptor mRNA expression levels. Additionally, it could upregulate genes that are responsible for steroid hormone metabolism and essential molecular and cellular functions [24,62].

Furthermore, ginsenoside Rg1, which structurally resembles steroid hormones and raises rats’ serum testosterone levels, is primarily responsible for the beneficial effects of dietary ginseng [65]. In [66] ginsenoside Rb1 (10 µg/kg bw) increased the secretion of LH by acting directly on the anterior pituitary gland. LH and FS play crucial roles in controlling testicular functions through their corresponding cognate receptors, the LH receptor (LHR) and the FSH receptor (FSHR) [67]. Similar to our findings, Refs. [68,69] found that testosterone concentrations and sperm populations were noticeably raised in comparison to the control through the addition of the PGE.

In view of the extended effects of feeding broodstock on their offspring, our results affirmed that larvae of broodstock fed a diet containing PGE had the best performance in comparison to those from broodstock not fed a diet containing PGE. All essential maternal reserves are transferred into the oocytes during the early stages of ovarian development. Thereafter, the provision of high-quality nutritive feed plays a significant role in the improvement of larval quality through increased survival, growth, and swim bladder inflation [70]. Therefore, up until the start of external feeding, the nutrient composition of the broodstock diet greatly influences the quality of the larvae. Some studies have demonstrated that the major reason for the positive effects of ginseng is the bioactive molecule ginsenoside [71,72]. In addition to its anti-inflammatory and anti-stress benefits, it possesses immunomodulatory and antioxidant qualities [73,74]. Ginseng polysaccharides have potential prebiotic effects, enriching beneficial gut microbiota and supporting intestinal health, which are critical for efficient nutrient absorption and disease resistance, leading to improved digestive enzyme activity, modulated intestinal microbiota, and enhanced immune responses in *Oreochromis niloticus* and *Carassius auratus* [75,76,77]. In addition, ginseng extracts can be utilized as a natural substitute for hygromycin as a growth promoter and immunoregulator in tilapia diets [77]. Ref. [58] discovered that the dietary supplementation of ginseng extracts greatly increased the survival rate of young *Oncorhynchus mykiss*.

## 5. Conclusions

It can be summarized that dietary PGE had positive effects on the reproductive physiology of Nile tilapia broodstock in a linear relationship as increasing its dose from 0.5 to 1.5 g/kg diet improved semen quality, blood androgens levels (testosterone and progesterone) and gonad morphometrics (egg diameter and the gonadosomatic index). These improvements lead to higher hatching rates, resulting in increased fry production and enhanced larval performance. Our results indicate that using *Panax ginseng* extract as a supplemental feed at a dose of 1.5 g/kg diet during the spawning season of Nile tilapia can boost reproductive efficiency and support sustainability. However, these findings highlight the need for further study to clarify the mechanisms, optimal dosages, feeding duration, and gender-specific responses.

## Figures and Tables

**Table 2:** Sperm quality of *Oreochromis niloticus* broodstock fed different doses of PGE-supplemented feed 30 days after starting the spawning experiment.

Items	Control	PGE0.5	PGE1	PGE1.5	±SEM	*p*-Value
pH	7.05 ^b^	7.12 ^ab^	7.16 ^ab^	7.23 ^a^	0.03	0.052
Sperm count, ×10^6^/mL	570 ^c^	618 b^c^	673 ^b^	735 ^a^	23.88	0.006
Motility, %	76.50 ^c^	84.00 ^b^	90.00 ^ab^	92.50 ^a^	2.43	0.012
Forward motility, %	31.50 ^c^	45.00 ^b^	48.50 ^ab^	52.50 ^a^	3.05	0.004
Sluggish motility, %	45.00	39.00	41.50	40.00	1.45	0.590
Immotile, %	23.50 ^a^	16.00 ^b^	10.00 ^bc^	7.50 ^c^	2.43	0.012

Significant differences are detected across means with different lowercase letters (*p* ≤ 0.05). PGE0.5, PGE1 and PGE1.5 are the tested doses of 0.5, 1, and 1.5 g of *Panax ginseng* extract per kg of diet.

**Table 3:** Blood biochemistry of *Oreochromis niloticus* females fed different doses of PGE-supplemented feed 30 days after the start and at the end of the spawning experiment.

Items	Control	PGE0.5	PGE1	PGE1.5	±SEM	*p*-Value
After 30 days from the start of the experiment						
Cortisol, µg/dl	25.00 ^a^	23.00 ^ab^	21.50 ^ab^	19.00 ^b^	0.93	0.050
Triglyceride, mg/dl	273.00 ^a^	258.00 ^b^	265.50 ^ab^	267.00 ^ab^	2.31	0.046
Cholesterol, mg/dl	244.50 ^a^	238.50 ^ab^	236.50 ^ab^	233.00 ^b^	1.79	0.041
Estrogen, pg/ml	3475 ^a^	3035 ^b^	2590 ^c^	2565 ^c^	141.62	<0.001
Progesterone, ng/ml	2.16 ^c^	2.91 ^b^	3.14 ^a^	3.24 ^a^	0.16	<0.001
After experimental duration (100 days)						
Cortisol, µg/dl	27.00 ^a^	25.50 ^a^	24.00 ^ab^	21.00 ^b^	0.91	0.036
Triglyceride, mg/dl	284.50 ^a^	269.50 ^b^	263.00 ^b^	260.00 ^b^	3.68	0.006
Cholesterol, mg/dl	245.50 ^a^	196.50 ^b^	190.50 ^bc^	184.00 ^c^	9.25	<0.001
Estrogen, pg/ml	3450 ^a^	3195 ^b^	3045 ^b^	3100 ^b^	61.69	0.016
Progesterone, ng/ml	1.91	2.09	2.31	2.51	0.22	0.875

Significant differences are indicated by different lowercase letters (*p* ≤ 0.05). PGE0.5, PGE1, and PGE1.5 are the tested doses of 0.5, 1, and 1.5 g of *Panax ginseng* extract per kg of diet.

**Table 4:** Blood biochemistry of *Oreochromis niloticus* males fed different doses of PGE-supplemented feed 30 days after the start and at the end of the spawning experiment.

Items	Control	PGE0.5	PGE1	PGE1.5	±SEM	*p*-Value
After 30 days from the start of the experiment						
Cortisol, µg/dl	28.00	26.50	25.50	24.00	0.73	0.296
Triglyceride, mg/dl	145.50 ^b^	158.50 ^ab^	163.50 ^a^	169.50 ^a^	3.60	0.035
Cholesterol, mg/dl	166.50 ^a^	149.00 ^b^	144.50 ^b^	153.00 ^ab^	3.42	0.045
Testosterone, ng/ml	6.46 ^b^	10.13 ^a^	10.74 ^a^	11.28 ^a^	0.75	0.016
After experimental duration (100 days)						
Cortisol, µg/dl	28.00	27.50	26.50	25.00	0.65	0.454
Triglyceride, mg/dl	148.00	149.00	150.50	157.50	1.81	0.254
Cholesterol, mg/dl	157.50 ^a^	145.00 ^b^	107.50 ^c^	101.50 ^c^	9.09	<0.001
Testosterone, ng/ml	3.95 ^c^	4.96 ^bc^	5.92 ^ab^	6.89 ^a^	0.44	0.024

Significant differences are indicated by different lowercase letters (*p* ≤ 0.05). PGE0.5, PGE1, and PGE1.5 are the tested doses of 0.5, 1, and 1.5 g of *Panax ginseng* extract per kg of diet.

**Table 5:** Reproductive efficiency of *Oreochromis niloticus* females that fed dietary PGE-supplemented feed for 100 days.

Items	Control	PGE0.5	PGE1	PGE1.5	±SEM	*p*-Value
Spawned female number/hapa/period	295.02 ^b^	339.48 ^ab^	355.99 ^a^	365.99 ^a^	10.29	0.046
Spawned female number/hapa/collection	33.39 ^b^	37.72 ^ab^	39.56 ^a^	40.67 ^a^	1.14	0.046
Spawned female, %/collection	37.10 ^b^	41.91 ^ab^	43.96 ^a^	45.19 ^a^	1.27	0.046
Number of spawning/female/periods	3.34 ^b^	3.77 ^ab^	3.96 ^a^	4.07 ^a^	0.11	0.047
Spawning intervals, days	29.95 ^a^	26.56 ^b^	25.32 ^b^	24.63 ^b^	0.83	0.034

Significant differences are detected across means with different lowercase letters (*p* ≤ 0.05). PGE0.5, PGE1, and PGE1.5 are the tested doses of 0.5, 1, and 1.5 g of *Panax ginseng* extract per kg of diet.

**Table 6:** Fry production of *Oreochromis niloticus* broodstock fed different doses of dietary PGE-supplemented feed for 100 days.

Items	Control	PGE0.5	PGE1	PGE1.5	±SEM	*p*-Value
Fry number per hapa/collection	11,245 ^b^	14,415 ^ab^	15,920 ^a^	16,515 ^a^	851.65	0.045
Fry number per female/collection	336.65 ^b^	381.73 ^ab^	401.88 ^a^	405.76 ^a^	11.37	0.049
Fry number per female/period	1124.50 ^b^	1441.50 ^ab^	1592 ^a^	1651.50 ^a^	85.17	0.050
Total hapa production, Fry/period	10,1205 ^b^	12,9735 ^ab^	14,3280 ^a^	14,8635 ^a^	7664.88	0.035
Feed required to produce 1000 fry, g	197.93 ^a^	162.59 ^b^	150.69 ^b^	148.15 ^b^	8.08	0.034

Significant differences are detected among means with different lowercase letters (*p* ≤ 0.05). PGE0.5, PGE1 and PGE1.5 are the tested doses of 0.5, 1, and 1.5 g of *Panax ginseng* extract per kg of diet.

**Table 7:** Larval performance of *Oreochromis niloticus* broodstock fed different doses of dietary PGE-supplemented feed for 100 days.

Items	Control	PGE0.5	PGE1	PGE1.5	±SEM	*p*-Value
Initial weight, mg/fish	10.45	10.55	10.65	10.75	0.05	0.069
Final weight, mg/fish	275.00 ^c^	277.50 ^bc^	283.00 ^ab^	286.00 ^a^	1.73	0.018
Weight gain, mg/fish	264.55 ^c^	266.95 ^bc^	272.35 ^ab^	275.25 ^a^	1.69	0.017
SGR, %/day	11.67 ^b^	11.68 ^b^	11.71 ^a^	11.72 ^a^	0.01	0.009
FCR, g feed/g gain	1.51 ^a^	1.50 ^a^	1.47 ^b^	1.45 ^b^	0.01	0.017
Survival rate, %	73.75 ^b^	76.49 ^a^	77.35 ^a^	77.67 ^a^	0.61	0.018

Significant differences are detected across means with different lowercase letters (*p* ≤ 0.05). PGE0.5, PGE1 and PGE1.5 are the tested doses of 0.5, 1, and 1.5 g of *Panax ginseng* extract per kg of diet.

## Data Availability

All data generated or analyzed during this study are included in this article.
